# Serum proteomics of severe fever with thrombocytopenia syndrome patients

**DOI:** 10.1186/s12014-022-09368-8

**Published:** 2022-08-13

**Authors:** Sang-Yeop Lee, Sung Ho Yun, Hayoung Lee, Yun Gyeong Lee, Giwan Seo, Nam Hoon Kim, Edmond Changkyun Park, Chang-Seop Lee, Seung Il Kim

**Affiliations:** 1 Research Center for Bioconvergence Analysis, Korea Basic Science Institute, Ochang, 28119 Republic of Korea; 2 Center for Convergent Research of Emerging Virus Infection, Korea Research Institute of Chemical Technology (KRICT), Daejeon, 34114 Republic of Korea; 3 Center for Research Equipment, Korea Basic Science Institute, Ochang, 28119 Republic of Korea; 4 Department of Bio-Analytical Science, University of Science and Technology, Daejeon, 34113 Republic of Korea; 5 Division of Healthcare and Artificial Intelligence, National Institute of Health, Osong, 28159 Republic of Korea; 6 Department of Internal Medicine, Jeonbuk National University Medical School, Jeonju, 54986 Republic of Korea; 7 Research Institute of Clinical Medicine of Jeonbuk National University–Biomedical Research Institute of Jeonbuk National University Hospital, Jeonju, 54907 Republic of Korea; 8 Critical Diseases Diagnostics Convergence Research Center, Korea Research Institute of Bioscience and Biotechnology, Daejeon, 34141 Republic of Korea

**Keywords:** SFTS, SFTSV, LC–MS/MS, Serum proteomics

## Abstract

**Background:**

Dabie bandavirus, also termed as severe fever with thrombocytopenia syndrome virus (SFTSV), was first isolated in China in 2010. At this time, the virus was found to have spread to South Korea, Japan, and other countries. A high case fatality rate is reported for SFTS, ranging from 12–50% within various sources. Several omics for clinical studies among SFTS patients as well as studies of cultured SFTSV have attempted to characterize the relevant molecular biology and epidemiology of the disease. However, a global serum proteomics analysis among SFTS patients has not yet been reported to date.

**Methods:**

In the current study, we evaluated comparative serum proteomics among SFTS patients (eight recovered patients and three deceased patients) with the goal of identifying the protein expression patterns associated with the clinical manifestations of SFTS.

**Results:**

The proteomic results in the current study showed that the coagulation factor proteins, protein S and protein C, were statistically significantly downregulated among the deceased patients. Downregulation of the complement system as well as prolonged neutrophil activation were also observed. Additionally, the downstream proteins of tumour necrosis factor alpha, neutrophil-activating cytokine, and interleukin-1β, an inflammatory cytokine, were overexpressed.

**Conclusions:**

Thrombocytopenia and multiple organ failure are the major immediate causes of death among SFTS patients. In this study, serum proteomic changes related to thrombocytopenia, abnormal immune response, and inflammatory activation were documented in SFTS patients. These findings provide useful information for understanding the clinical manifestations of SFTS.

**Supplementary Information:**

The online version contains supplementary material available at 10.1186/s12014-022-09368-8.

## Background

Severe fever with thrombocytopenia syndrome (SFTS) was first reported in 2009. This syndrome is caused by Dabie bandavirus, also termed as severe fever with thrombocytopenia syndrome virus (SFTSV). SFTSV was first isolated in China in 2010, where it was identified as belonging to the family *Phenuiviridae* family [1]. SFTS was first reported in Japan in 2012 and was first repoted in South Korea in 2013. Though the average fatality rate varies between different regions, the reported fatality rate for SFTS is relatively high overall, ranging from 12 to 50% [2–4]. Patients with SFTS present with high fever followed by thrombocytopenia, leukocytopenia, and liver injury. Cases of fatality typically present with symptoms of disseminated intravascular coagulation (DIC) as well as multiple organ failure [5, 6]. In connection with these clinical symptoms, biomarker levels within liver function tests, including serum aspartate aminotransferase (AST), alanine aminotransferase (ALT), and lactic acid dehydrogenase (LDH), are typically high. Likewise, the activated partial thromboplastin time (aPTT) is typically prolonged in the multiple organ failure stage in patients with SFTS [7]. Additionally, inflammatory cytokine levels are also elevated in patients with SFTS. A previous report confirmed that tumour necrosis factor (TNF)-α, interferon (IFN)-α, interleukin 6 (IL-6), and interleukin 10 (IL-10) expression is higher in patients with SFTS as compared with healthy individuals [8, 9]. TNF-α, INF-α and IL-6 levels are highly associated with clinical severity.

Over the past decade since the isolation of SFTSV was reported, various approaches, including clinical analysis and cytokine production analysis, have been used to identify the pathogenesis of SFTSV. Although omics technology presents highly effective methodology for pathogenic investigation, omics studies of SFTSV or SFTS patients have only recently been reported [10–13]. Patient age is an important known risk factor for SFTS disease severity. For example, a ferret model analysis confirmed that aged ferrets infected with SFTS have symptoms such as severe thrombocytopenia, high fever, and reduced white blood cell counts. In a transcriptomic analysis, interferon-mediated anti-viral signalling was strongly observed in young ferrets, and inflammatory response-related genes were upregulated in aged ferrets [10]. In a proteomic study of SFTSV-infected cells, the unfolded protein response of host cells was found to play an important role in SFTSV infection [11]. Another proteomic study of SFTSV-infected cells reported that mitochondrial DNA was released upon SFTSV infection, inducing *NLRP3* inflammasome activation [12]. In addition to these host response mechanism studies, serum immune profiling among patients with SFTS as well as single-cell RNA-seq studies of peripheral blood mononuclear cells (PBMCs) report that overexpressed inflammatory cytokines are related to disease severity among patients with SFTS [13]. These studies provide valuable information with respect to SFTSV infection in vivo and in vitro.

Blood biomarkers are important for clarifying disease status. However, a global proteomic analysis of patient sera is yet to be reported among SFTS patients. Therefore, in this study, we performed serum proteomics in SFTS patients to identify the proteins associated with the clinical manifestations of the disease as well as to characterize changes according to disease severity. To the best of our knowledge, this is the first clinical study to examine proteomic results in sera among SFTS patients.

## Methods

### Patients and clinical samples

Sera were isolated from eleven SFTS patients admitted to Jeonbuk National University Hospital. Serum samples were obtained from blood within six hours following confirmation of infectious with the SFTS virus. Laboratory-confirmed SFTS virus infection was defined according to the following criteria: (1) clinical symptoms (e.g., fever), (2) epidemiological evidence supporting the possibility of a tick bite, and (3) identification of viral RNA through reverse transcription polymerase chain reaction (RT–PCR). To detect SFTSV RNA, RNA was extracted from the serum using a QIAamp Viral RNA Mini Kit (Qiagen, Hilden, Germany) according to manufacturer instructions. One-step RT–PCR was performed using a ToPscript™ One-step RT PCR DryMix (Enzynomics, Daejeon, Korea) with the primers MF3 (5′- GATGAGATGGTCCATGCTGATTCT-3′) and MR2 (5′- TCATGGGGTGGAATGTCCT CAC-3′).

### Proteomic analysis via LC–MS/MS

Liquid chromatography with tandem mass spectrometry (LC–MS/MS) analysis was performed according to the methodology presented in a previous report [14]. Briefly, the total extracted proteins were separated by 12.5% sodium dodecyl sulphate–polyacrylamide gel electrophoresis (SDS-PAGE) and were then subjected to in-gel tryptic digestion. Chemical contaminants in the tryptic peptide mixture were cleaned using an MGU30-18 trapping column (LC Packings). The peptides eluted from the column were directed into a 10 cm × 75 μm IDC18 reverse phase column (PROXEON, Odense, Denmark) at a flow rate of 300 nL/min. Peptides were eluted with a gradient of 0–65% acetonitrile over the course of 80 min. A Q Exactive Plus mass spectrometer (Thermo Scientific, Waltham, MA, USA) was used for tandem mass spectrometry (MS/MS) conducted in data-dependent mode. Each full mass spectrometry (MS) scan (m/z range 400–2000) was followed by three MS/MS scans confirming the most abundant precursor ions in the mass spectrum.

### Public SFTS patient dataset

Proteomic results provide useful information for elucidating biological processes mediating disease aetiology based on proteome levels. However, proteomic analyses require complementary omics tools to confirm large-scale changes. Proteomics alone can only give limited information with respect to differentially expressed proteins (DEP). Therefore, we performed additional analysis using transcriptomic data downloaded from the U.S. National Center for Biotechnology Information (NCBI) Sequencing Read Archive (SRA; https://www.ncbi.nlm.nih.gov/sra). We re-analysed the GSE144358 dataset subjected to RNA-seq. analysis using human serum mRNA from SFTS patients [12]. The following raw FASTQ files for the GSE144358 dataset were downloaded from the Gene Omnibus Expression (GEO) Accession: GSM4286781 to GSM4286858. This dataset contains 21 healthy controls, 15 recovered SFTS patients, and 19 deceased SFTS patients. Differentially expressed genes (DEG) were identified using the DESeq2 R package (The R Project for Statistical Computing, Vienna, Austria) [15] with hg38 as the reference sequence.

### Statistical and bioinformatic analyses

We performed Student’s *t*-test to evaluate univariate associations using R statistical package. Ingenuity pathway analysis (IPA) was used for network analysis and enrichR [16] was used for the functional analysis of DEPs. Protein quantification was identified through Maxquant 2.6.1 (Munich, Germany) with the Uniprot Human proteome sequence (UP000005640) as reference database [17]. The protein search parameter used the orbitrap default condition and the false discovery rate was set to 0.01. The DEPs were calculated in Persous 1.6.2 (a software platform within Maxquant) using the label-free quantification (LFQ) values generated from Maxquant as input data [18]. The cut-off value of DEP was determined to be < 0.05 for *t*-test p-values and fold changes > 2 (|fold-change|> 2).

### Ethics approval

Serum samples were collected from subjects according to registered protocols approved by the Institutional Review Board (IRB) of Jeonbuk National University Hospital, and all patients provided their written informed consent prior to participation (IRB registration number 2019-06-020). This study was conducted in accordance with the principles of the Declaration of Helsinki.

## Results and discussion

### Evaluation of clinical samples

Sera were isolated from eleven patients with SFTS who were hospitalized in the Jeonbuk National University Hospital from 2018 to 2019. There were no statistically significant differences in leukocyte and platelet counts in the eleven enrolled patients at the time of serum isolation. However, among the enrolled patients, three patients ultimately died. These patients had statistically significantly higher levels of AST, ALT, L-lactate dehydrogenase (LD), creatinine, and high-sensitivity C-reactive protein (hs-CRP). Additionally, the aPTT in SFTS patients was prolonged than as compared with the normal range. Patients with SFTS are generally known to die of organ failure with thrombocytopenia. However, in the case of the deceased patients evaluated in this study, no statistically significant decreases in platelet counts were observed. We found that the aPTT was prolonged in the deceased patients as compared to that in the recovered patients. In this study, we aimed to identify several factors relating to death caused by SFTSV infection using a serum proteomics analysis. The detailed clinical characteristics of the enrolled patients are described in Table 1.

**Table 1:** Demographic, clinical characteristics and laboratory findings of SFTS patients

Characteristics	Recovered (n = 8)	Deceased (n = 3)	*p-value*
Epidemiology, no. (%)			
Age, mean Y ± SD (range)	71.0 ± 12.4	69.3 ± 11.02	0.8429
Female	4 (50.0)	2 (66.7)	0.9103
Occupational exposure	3 (37.5)	1 (33.3)	0.6618
Comorbidities, no. (%)			
Cardiovascular disease^*^	1 (12.5)	0	0.5683
Cerebrovascular disease	1 (12.5)	0	0.5683
Pulmonary disease^**^	0	0	–
Chronic kidney disease	0	0	–
Diabetes mellitus	1 (12.5)	0	0.5683
Malignancies	0	0	–
Clinical signs and symptoms, no. (%)			
Headache	2 (25.0)	1 (33.3)	0.8075
Dyspepsia	5 (62.5)	2 (66.7)	0.9103
Nausea/vomiting	5 (62.5)	1 (33.3)	0.4385
Abdominal pain	5 (62.5)	0	0.0738
Chills	6 (75.0)	3 (100)	0.3893
Myalgia	5 (62.5)	2 (66.7)	0.9103
Fatigue	7 (87.5)	1 (33.3)	0.4761
Rash/Eschar	3 (37.5)	1 (33.3)	0.9103
Tick bite	6 (75.0)	1 (33.3)	0.2413
Laboratory values, median (IQR)			
WBC count, × 1000/mm^3^	2276.3 (1150–4150)	1866.7 (1240–2400)	0.5659
Platelet count, × 1000/mm^3^	57,125 (25,000–93,000)	33,000 (21,000–45,000)	0.1286
aPTT, sec	40.9 (33.6–52.4)	84.4 (78.5–90.9)	** < 0.001**
Total bilirubin, mg/dL	1.5 (0.58–5.13)	2 (1.08–2.5)	0.5740
Albumin, g/dL	3.2 (2.8–3.5)	2.5 (1.9–2.9)	** < 0.001**
AST, IU/L	337.4 (71–1210)	6670.7 (4710–9956)	** < 0.001**
ALT, IU/L	121.8 (46–242)	1096.3 (846–1366)	** < 0.001**
LD, IU/L	1562.6 (775–3082)	16,619.7 (14,915–18,030)	** < 0.001**
Creatinine, mg/dL	0.9 (0.44–1.28)	2.4 (2.22–2.8)	** < 0.001**
hs-CRP, mg/dL	9.5 (1.15–22.4)	34 (32.33–36.31)	** < 0.001**
Ct value			
S segment	31.1 (23.56–36.88)	25.4 (21.16–31.5)	0.0805
M segment	30.1 (22.26–33.65)	26.8 (22.26–33)	0.2839

*SFTS* severe fever with thrombocytopenia syndrome; *SD* standard deviation; *IQR*, interquartile range; *WBC* white blood cell; *aPTT* activated partial thromboplastin time; *AST* aspartate aminotransferase; *ALT* alanine aminotransferase; *LD* lactate dehydrogenase; *CRP* C-reactive protein

*Includes myocardial infarction, congestive heart failure, and peripheral vascular disease

**Chronic obstructive pulmonary disease, asthma

Statistically significant values are shown in bold

### Proteomic analysis

**Fig. 1:**
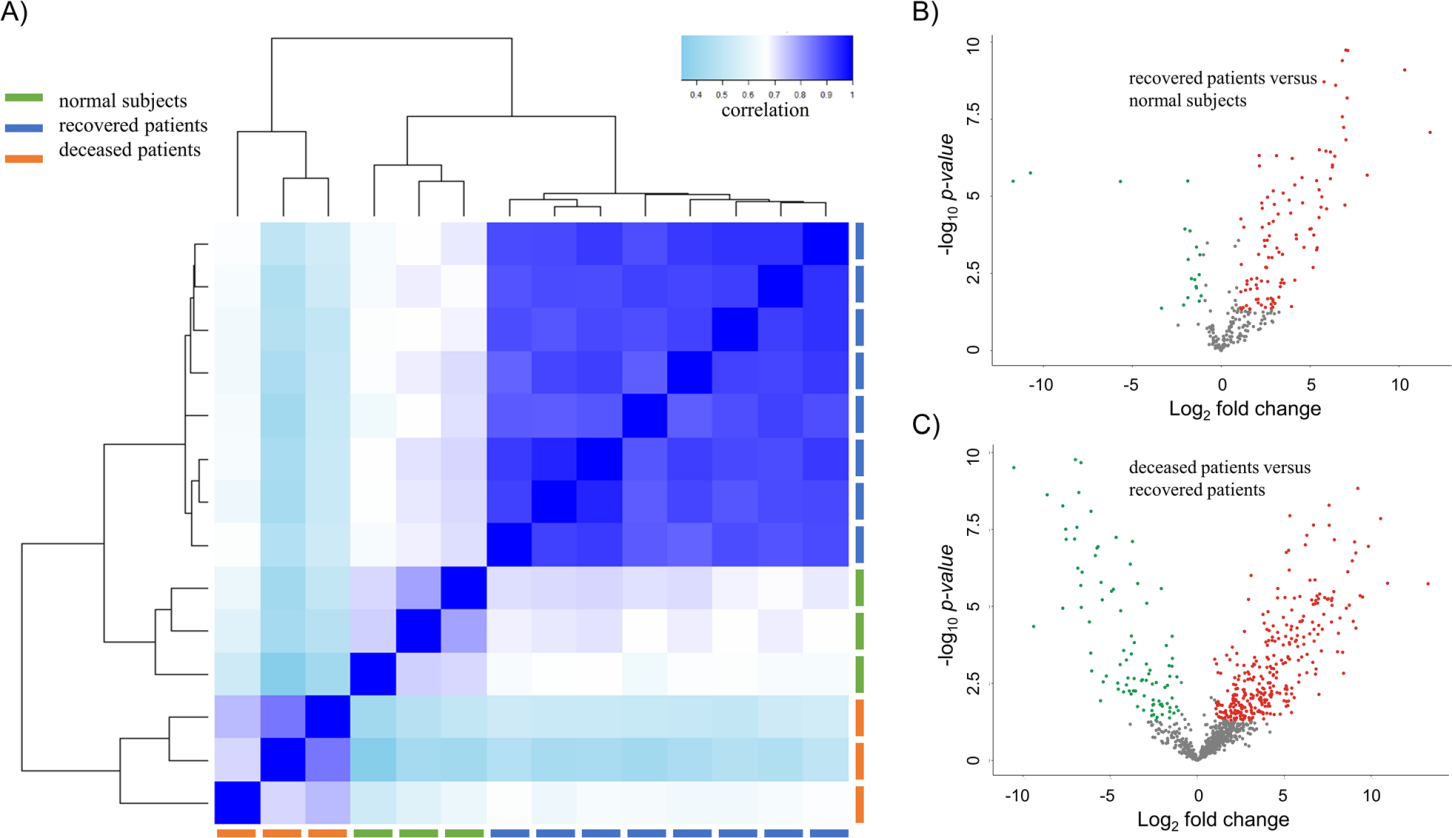
Summary of the proteomic analysis among severe fever with thrombocytopenia syndrome (SFTS) patients. **A** A sample correlation heatmap showed that proteome results were clearly divided into three clusters, according to each group (recovered patients, deceased patients, and normal controls). **B**, **C** Red dots and green dots indicate overexpressed and downregulated proteins, respectively. Over 40% of the proteins were identified to be differentially expressed (fold change ≥ 2, p-value < 0.05) in the deceased patients as compared with the recovered patients

Proteomic analysis was performed via LC–MS/MS on sera from eleven SFTS patients and three normal subjects. We classified the SFTS patients into two groups: a recovered patient group (eight patients), and a deceased patient group (three patients). The sample correlation heatmap of the total proteomics data showed that the three groups were clearly divided into three clusters (Fig. 1A). The normal subjects and the recovered patients were more closely clustered than the deceased patients. A comparison of protein quantification results between the recovered patients and the normal subjects showed that 85 proteins were overexpressed and 17 proteins were downregulated in the recovered patients (Fig. 1B and Additional file 1: Table S1). In comparing between the deceased patients and the recovered patients, 315 proteins were overexpressed, and 91 proteins were downregulated in the deceased patients (Fig. 1C and Additional file 1: Table S1). A total of 406 proteins (43.3%) among the 937 identified proteins from the deceased patients were differentially expressed when compared with the recovered patients. However, when comparing the recovered patients with the normal subjects, we confirmed that only 20.9% of the proteins identified in the recovered patients were differentially expressed. Therefore, characterizing DEPs in the deceased patients as compared with that in recovered patients may help elucidate the phenomenon of severity in patients with SFTS.

### Comparative analyses of canonical pathways

**Fig. 2:**
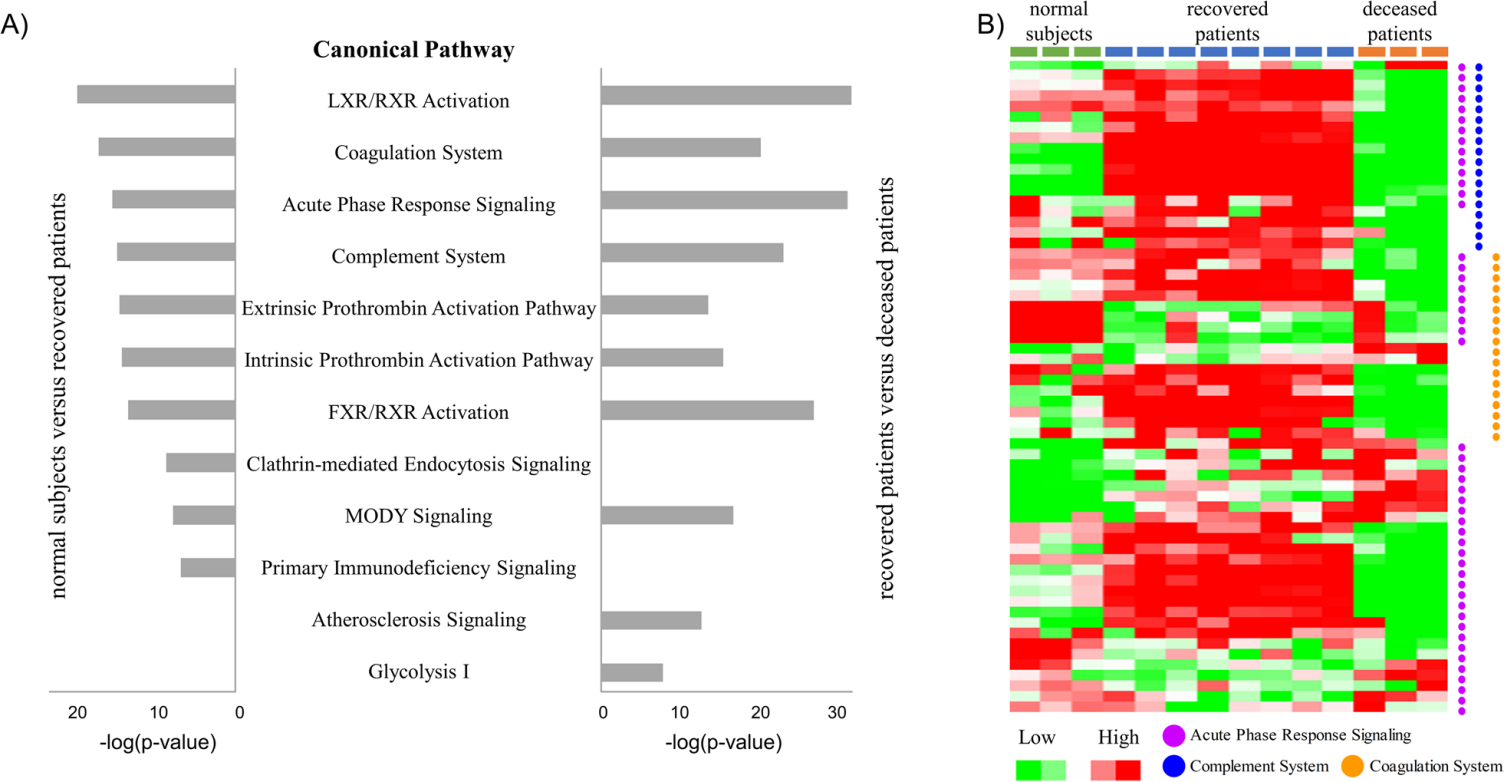
Analysis of enriched canonical pathways in severe fever with thrombocytopenia syndrome (SFTS) patients. **A** Eight canonical pathways were commonly involved in the two comparative conditions (normal subjects versus recovered patients, recovered patients versus deceased patients). Among these pathways, the coagulation system, acute phase response signalling, and the complement system were evaluated in detail. **B** The protein expression heatmap showed that the proteins within the three pathways tended to be overexpressed in recovered patients

To identify the key molecular signature among patients with SFTS, we performed an enriched pathway analysis. Specifically, the enriched canonical pathways were investigated using an IPA analysis of DEP according to the prognosis of patients with SFTS (Fig. 2A). Among the identified canonical pathways, we were most interested in three canonical pathways (the acute phase response, the coagulation system, and the complement system), all of which were detected in both comparative conditions (normal subjects versus recovered patients and recovered patients versus deceased patients) (Fig. 2A). The proteins associated with the three pathways tended to be overexpressed in the recovered patients and downregulated in the deceased patients (Fig. 2B and Table 2).

**Table 2:** Differentially expressed proteins in SFTS patients

Uniprot_Id	Description	Gene	Recovered patients/normal subjects		Deceased patients/recovered patients	
			*p-value*	log2FC	*p-value*	log2FC
*Complement system*						
P09871	Complement C1s subcomponent	C1S	1.0E−05	5.666	2.2E−07	− 5.847
P13671	Complement component C6	C6	3.2E−06	− 1.882	0.0031	− 4.564
P10643	Complement component C7	C7	0.0168	− 1.125	0.0008	− 4.422
A0A3B3ISR2	Complement subcomponent C1r	C1R	2.2E−05	5.615	7.9E−06	− 2.897
P0C0L5	Complement C4-B	C4B	0.0092	0.867	0.0002	− 1.774
P0C0L4	Complement C4-A	C4A	4.8E−07	2.138	0.0001	− 1.44
P02748	Complement component C9	C9	0.4626	− 0.243	0.024	− 1.089
P11215	Integrin alpha-M	ITGAM			0.4126	− 0.598
Q5SR44	Complement receptor type 1	CR1			0.1442	1.494
O00187	Mannan-binding lectin serine protease 2	MASP2	0.0048	1.702	0.0323	4.416
*Coagulation system*						
A0A3B3ISJ1	Vitamin K-dependent protein S	PROS1	1.7E−05	2.286	1.7E−10	− 6.992
E7END6	Vitamin K-dependent protein C	PROC	0.0008	2.107	1.4E−05	− 4.393
P05546	Heparin cofactor 2	SERPIND1	0.716	− 0.111	0.0021	− 3.98
P01023	Alpha-2-macroglobulin	A2M	0.4494	0.121	0.0026	− 3.198
P12259	Coagulation factor V	F5	0.1737	1.225	0.0159	− 2.542
P04275	von Willebrand factor	VWF	0.5524	0.697	0.1201	− 1.945
P00740	Coagulation factor IX	F9	0.0348	1.425	0.0194	− 1.564
P01009	Alpha-1-antitrypsin	SERPINA1	0.4545	− 0.447	0.8891	0.092
P01008	Antithrombin-III	SERPINC1	0.2133	− 0.525	0.4596	0.272
P08697	Alpha-2-antiplasmin	SERPINF2	0.0001	− 2.042	0.273	0.541
P05154	Plasma serine protease inhibitor	SERPINA5	0.8133	− 0.168	0.0001	5.42
*Downstream target of TNF-α and IL-1β*						
P05109	Protein S100-A8	S100A8	0.0065	3.536	0.0445	3.82
P06702	Protein S100-A9	S100A9	0.0005	4.66	0.0708	2.681
P02647	Apolipoprotein A-I	APOA1	0.0077	− 0.859	0.0003	− 3.788
P02649	Apolipoprotein E	APOE	0.0525	0.935	1.1E−07	− 5.696

The complement system is the first innate immune defence against viral infections. However, in the deceased patients, the component proteins of the complement system (except for MASP2) were downregulated when compared to the recovered patients (Fig. 2B and Table 2) and the normal subjects (data not shown). We infer that the complement system did not seem to be activated in the deceased patients. However, the complement proteins C4A, C4B, C1S, and C1R were upregulated in the recovered patients as compared to in the normal subjects (Table 2). Further, the expressions of complement C6 and C7 proteins were decreased in recovered patients as compared to the normal subjects (Table 2). Many viruses have been reported to evade host immune responses through complement evasion strategies [19]. However, to the best of our knowledge, a complement evasion strategy has not been reported for SFTSV, which belongs to the *Phlebovirus* genus. The current study provides clues that there is a possibility of complement system avoidance in SFTSV, which was unknown until now. Therefore, additional analysis is necessary to confirm the complement evasion strategies within SFTSV.

Protein expression in the coagulation system, another top-ranked canonical pathway, changed dramatically according to patient group in the current study (Fig. 2B and Table 2). Among the coagulation-related proteins, protein S, protein C, coagulation factor V, and coagulation factor IX were overexpressed in recovered patients (Table 2). Though the aPTT of recovered patients (33.6–52.4 s) was longer than the normal range (21.0–35.0 s), the upregulation of protein C suggests that the anticoagulation system remains functional in recovered patients. In contrast, drastic downregulation of coagulation proteins and protein C were found in deceased patients. The coagulation system is an important mechanism mediating the innate immune response and thrombocytopenia [20],which is of vital importance as thrombocytopenia is one of the most critical factors in mortality outcomes among patients with SFTS [5, 6]. In addition, proteins C and S are major proteins that play important roles in the anticoagulant system [21]. The downregulation of coagulation proteins and protein C among deceased patients may occur due to liver dysfunction observed in the deceased patients [22]. Therefore, we can assume that the coagulation system proteins had been consumed during SFTSV infection in the deceased patients. The highly prolonged aPTT time (78.5–90.0 s) and the inactivation of the coagulation system consistently seen in deceased patients suggest that the thrombosis system is already in a DIC state.

In conclusion, we observed that the initial immune response (i.e., the complement system) did not properly respond to the SFTSV infection process among the deceased patients. Additionally, we observed that the patients progressed to the DIC state due to the dysfunction of the coagulation system.

### Differentially expressed proteins

**Fig. 3:**
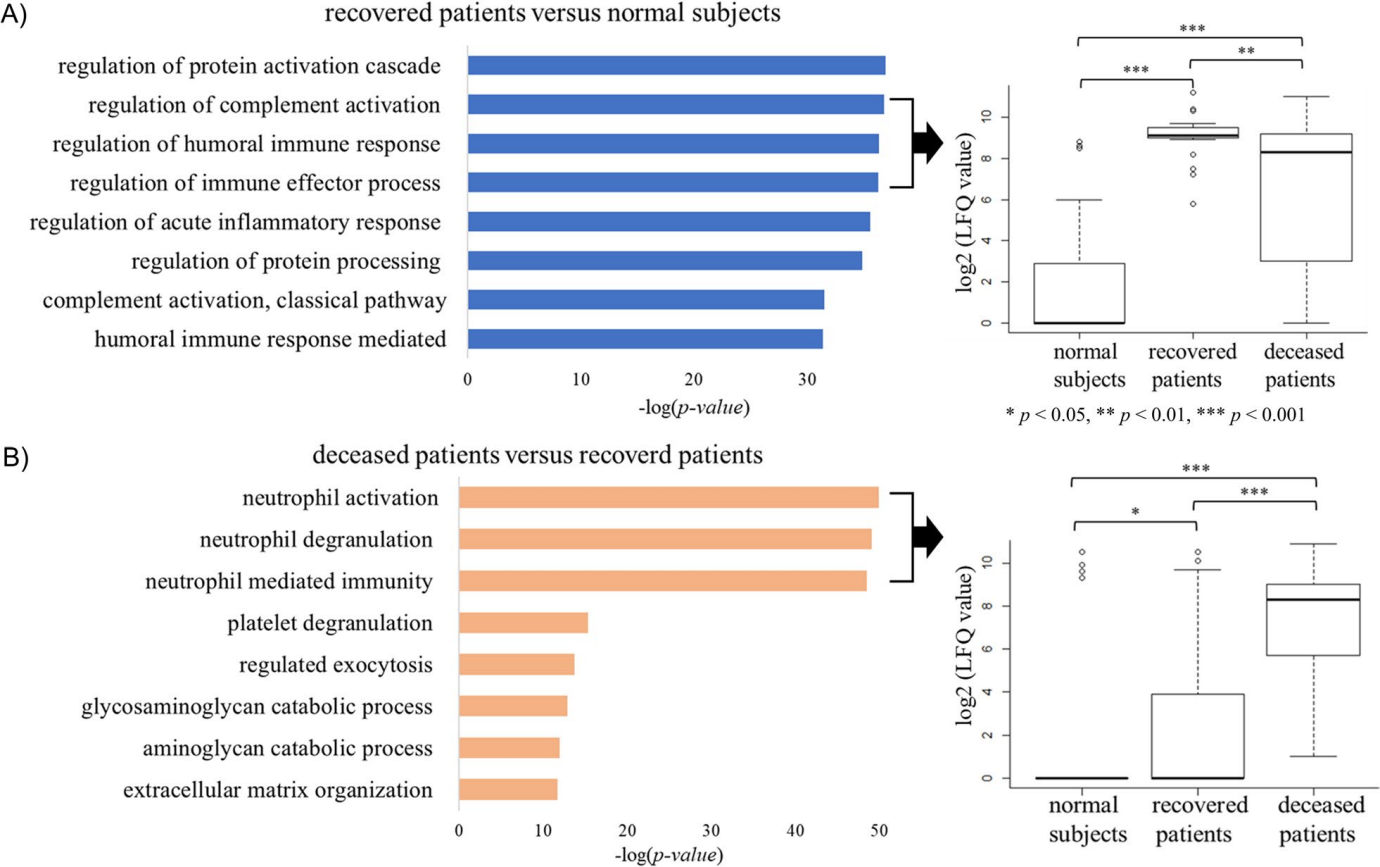
Results of the Gene Ontology Enrichment Analysis. **A** Comparison of protein expression between recovered and normal subjects. Immune regulation-related terms are highly ranked in the gene ontology enrichment analysis. The expression levels of proteins included in these terms are significantly overexpressed in recovered patients compared to normal subjects. **B** Comparison of protein expression between deceased and recovered patients. Neutrophil-associated proteins are dominant in deceased patients, and these proteins are overexpressed in deceased patients compared to recovered patients and normal subjects

**Fig. 4:**
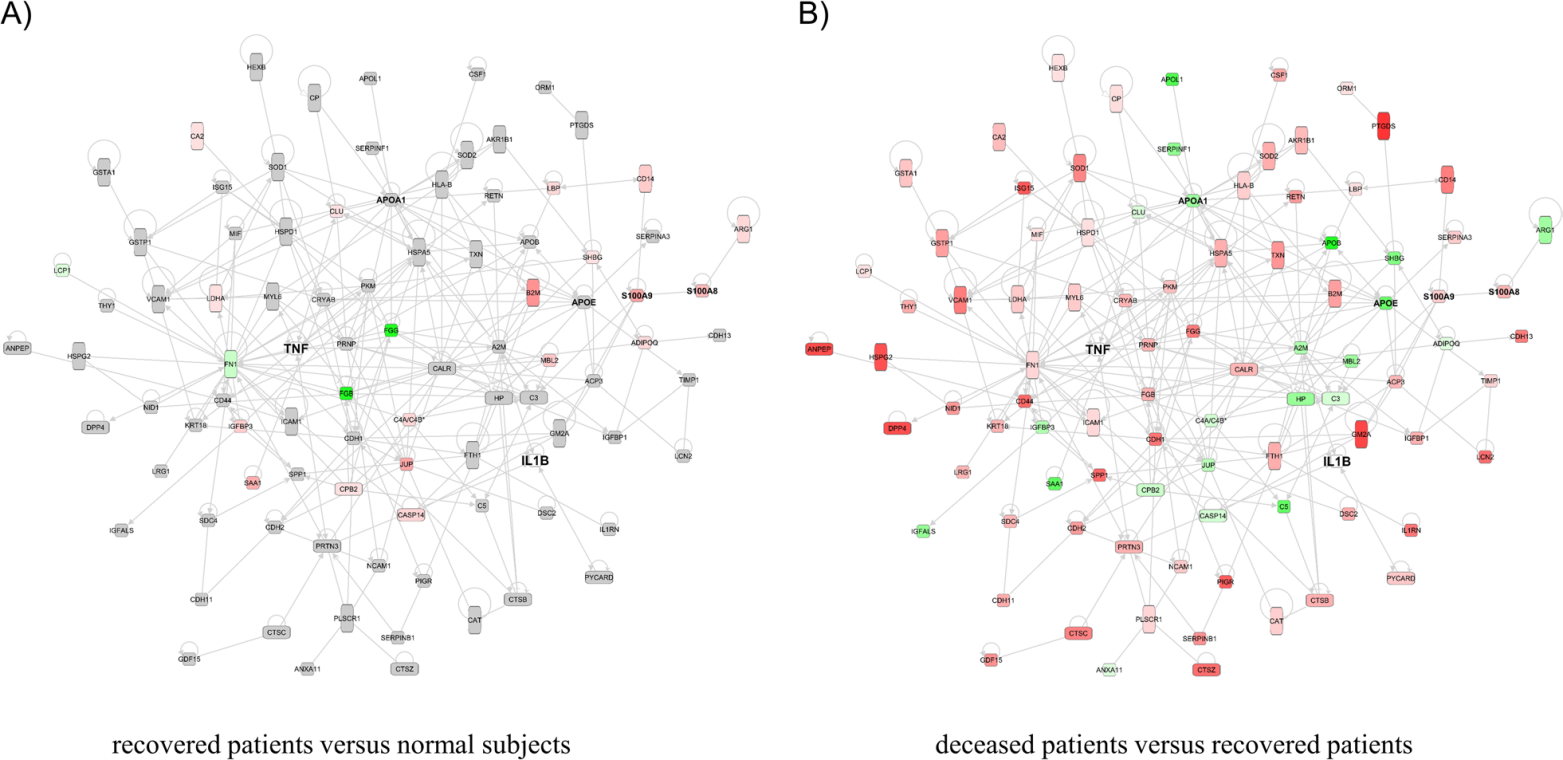
Gene network of downstream target proteins of TNF-α and IL-1β in SFTS patients. Comparative analysis of the downstream target proteins of TNF-α and IL-1β between recovered patients and normal subjects (**A**) and between deceased patients and recovered patients (**B**). The red, green, grey, and blank boxes indicate overexpressed, downregulated, non-statistically significantly altered, and unidentified proteins, respectively. IL-1ß, interleukin 1ß; SFTS, severe fever with thrombocytopenia syndrome; TNF, tumour necrosis factor

The GO enrichment test was performed to evaluate DEP between the comparison groups with the goal of identifying the biological processes present in SFTS patients. The top eight biological processes in each comparison group are summarized in Fig. 3. In comparing between the normal subjects and the recovered patients, we identified immune regulation-related proteins as highly ranked biological processes (Fig. 3A). Conventional medical wisdom indicates that a host’s immune system responds during SFTSV infection, thus resulting in subsequent recovery. However, the GO enrichment test results comparing between recovered patients and deceased patients in the current study revealed that neutrophil activation-related processes occur at a much higher rate within deceased patients (Fig. 3B and Additional file 1: Table S1). White blood cell (WBC) counts among SFTS patients were lower than the normal range. However, the WBC count was not statistically significantly different between recovered and deceased patients (Table 1). Nevertheless, all the proteins involved in the neutrophil activation-related processes (i.e., neutrophil activation, neutrophil degranulation, and neutrophil-mediated immunity) among the deceased patients were overexpressed as compared to the recovered patients. Neutrophils are considered the first immune responders to invading infectious pathogens [23]. However, long-term activation of neutrophils damages host cells, and is thus known to be harmful to patients [24]. Cytokines expressed in neutrophils have various activities associated with immune response and inflammation [25]. Therefore, serum proteomics using IPA upstream regulatory analysis was performed in the current study, confirming the presence of proteins that are influenced by cytokines. Our results suggest that TNF-α and interleukin 1 beta (IL-1β) are the top upstream regulators in SFTS patients. TNF-α is one of the essential cytokines inducing neutrophil activation and IL-1β is the major pro-inflammatory cytokine secreted from neutrophils [26]. Therefore, we confirmed the expression of TNF-α and IL-1β in the current study. Although TNF-α and IL-1β were not detected in the proteomic analysis, TNF-α and IL-1β have been reported to be induced in SFTS patients [8]. In addition, the proteomic results in our study confirmed changes in the expression of the downstream target proteins TNF-α and IL-1β between patient groups (Fig. 4 and Additional file 1: Table S1). Among these proteins, S100A8 and S100A9 were statistically significantly overexpressed and apolipoprotein A-I and apolipoprotein E were statistically significantly downregulated in SFTS patients (Table 2). S100A8 and S100A9 are expressed in neutrophils and monocytes and play an important role in stimulating leukocyte recruitment, inducing cytokine secretion, and regulating inflammatory processes. S100A8 and S100A9 were overexpressed to a higher degree in the deceased patients as compared with the recovered patients, indicating that inflammatory processes were more activated in the deceased patients. In contrast, the expression of APOA1 and its regulating protein APOE, which has an anti-inflammatory function, was reduced in the deceased patients (Table 2) [27]. Most other downstream target proteins did not show noticeable differences in protein expression levels in the recovered patients as compared with the normal subjects (Fig. 4A). However, the level of expression of proteins regulated by TNF and IL-1β was higher in the deceased patients (Fig. 4B and Additional file 1: Table S1). These results suggest that, in the deceased patients, inflammatory processes were overactivated by neutrophil activation.

**Fig. 5:**
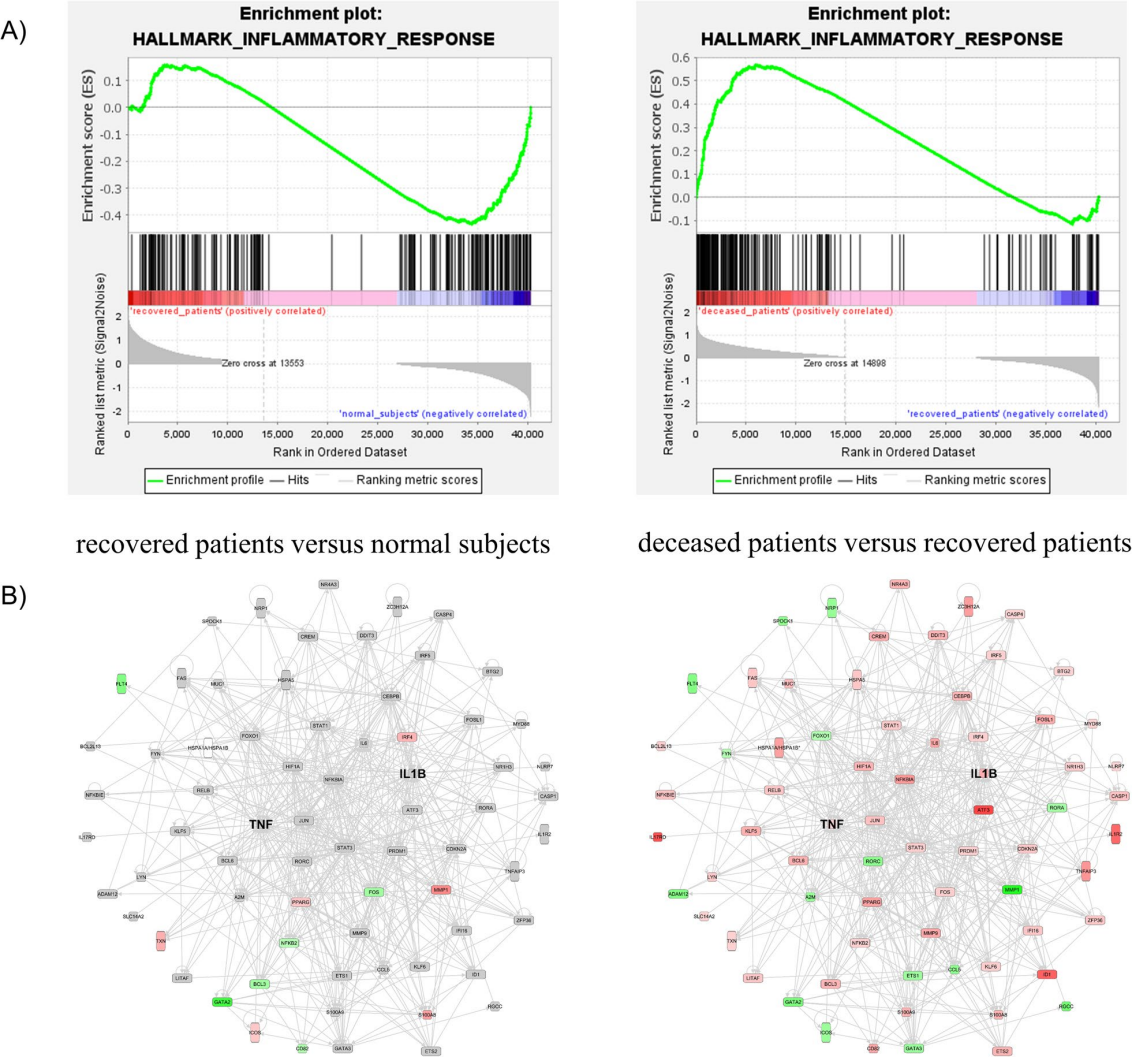
Gene set enrichment and gene network analysis using transcriptomics data among SFTS patients. Enrichment plots of inflammatory response genes in recovered patients and deceased patients are shown in the top portion of the figure (**A**). The lower half portion of the figure shows the results of gene network analyses with respect to TNF-α and IL-1β in recovered and deceased patients (**B**). IL-1ß, interleukin 1ß; SFTS, severe fever with thrombocytopenia syndrome; TNF, tumour necrosis factor

Recently, Li et al. [12] reported the results of a transcriptomic study among SFTS patients. We used the raw transcriptomic data for our gene set enrichment analysis and found that inflammatory response genes exhibited higher activation among deceased patients (Fig. 5A). Gene network analysis for the downstream targets of TNF-α and IL-1β revealed that the related genes were significantly overexpressed in the deceased patients. These results correlate with our proteomic findings (Fig. 5B).

## Conclusion

SFTSV is a novel phlebovirus recently reported and characterized in East Asia [1]. Many aspects with respect to the viral life cycle, infection mechanisms, and pathogenicity of SFTS/SFTSV remain unknown. In this study, we performed serum proteomics among SFTS patients, which were comparatively analysed to identify host responses on the proteomic level. The small sample size is a limitation of this study. We confirmed proteomic results in serum RNA-Seq from SFTS patients using a public database to overcome this limitation. However, further studies are needed to confirm protein expression changes in larger samples of SFTS patients. Despite this limitation, the study revealed an association between the serum proteomic changes and the clinical manifestations of SFTS. The clinical symptoms did not significantly differ between the deceased and recovered patients except for aPTT and liver function (Table 1). Moreover, the mental status of the deceased patients tended to be drowsier than that of the recovered patients. However, the serum samples of patients with SFTS were collected at similar times after symptom onset (Additional file 2: Figure S1). Although there were some deviations, samples from two of the three deceased patients were collected at 1 and 2 days after symptom onset, while the sample from the remaining one patient was collected 10 days later. The samples from the recovered patients were obtained 2–8 days after symptom onset. Therefore, the sample collection time for deceased patients was before the exacerbation of SFTS symptoms. Serum proteomics among SFTS patients revealed that the deceased patients had a problematic innate immune response, including with respect to the complement system as well as prolonged neutrophil activation and were more likely to develop progressive DIC due to dysfunction of the coagulation system. Additionally, inflammatory proteins that are downstream targets of TNF-α and IL-1β were found to be overexpressed in fatal cases of SFTS. There have been previous reports of transcriptomics and cytokine assays among serum samples from SFTS patients. However, to our knowledge, there have been no previous reports of serum proteomic studies in SFTS patients. Therefore, these findings provide useful information for understanding the clinical manifestations of SFTS.

## Supplementary Information

**Additional file 1: Table S1.** Results of serum proteomic analyses among severe fever with thrombocytopenia syndrome severe fever with thrombocytopenia syndrome (SFTS) patients.

**Additional file 2: Figure S1.** The hospitalization timeline for patients with SFTS.

## Data Availability

All data generated during this study are included in this published article. Total list of identified proteins has been uploaded as additional file. The mass spectrometry proteomics data have been deposited to the ProteomeXchange Consortium (Dataset identifier: PXD033989).

## Abbreviations

aPTT: Activated partial thromboplastin time
ALT: Alanine aminotransferase
AST: Aspartate aminotransferase
DEG: Differentially expressed genes
DEP: Differentially expressed proteins
DIC: Disseminated intravascular coagulation
GEO: Gene Omnibus Expression
hs-CRP: High-sensitivity C-reactive protein
IFN: Interferon
IL-1ß: Interleukin 1ß
IL-6: Interleukin 6
IL-10: Interleukin 10
IPA: Ingenuity pathway analysis
IRB: Institutional review board
LC–MS/MS: Liquid chromatography with tandem mass spectrometry
LD: L-lactate dehydrogenase
LDH: Lactic acid dehydrogenase
MS: Mass spectrometry
MS/MS: Tandem mass spectrometry
NCBI: National Center for Biotechnology Information
PMBCs: Peripheral blood mononuclear cells
RT-PCR: Reverse transcription polymerase chain reaction
SDS-PAGE: Sodium dodecyl sulphate–polyacrylamide gel electrophoresis
SFTS: Severe fever with thrombocytopenia syndrome
SFTSV: Severe fever with thrombocytopenia syndrome virus
SRA: Sequencing Read Archive
TNF: Tumour necrosis factor
