# Supplementary material for: Serum proteomics of severe fever with thrombocytopenia syndrome patients
Source: Clin Proteomics. 2022 Aug 13;19:32. doi: 10.1186/s12014-022-09368-8 (PMC9375430; doi:10.1186/s12014-022-09368-8)
Supplement: Supplementary file 2 — Additional file 2: Figure S1. The hospitalization timeline for patients with SFTS. [file 12014_2022_9368_MOESM2_ESM.pptx]

## Slide 1
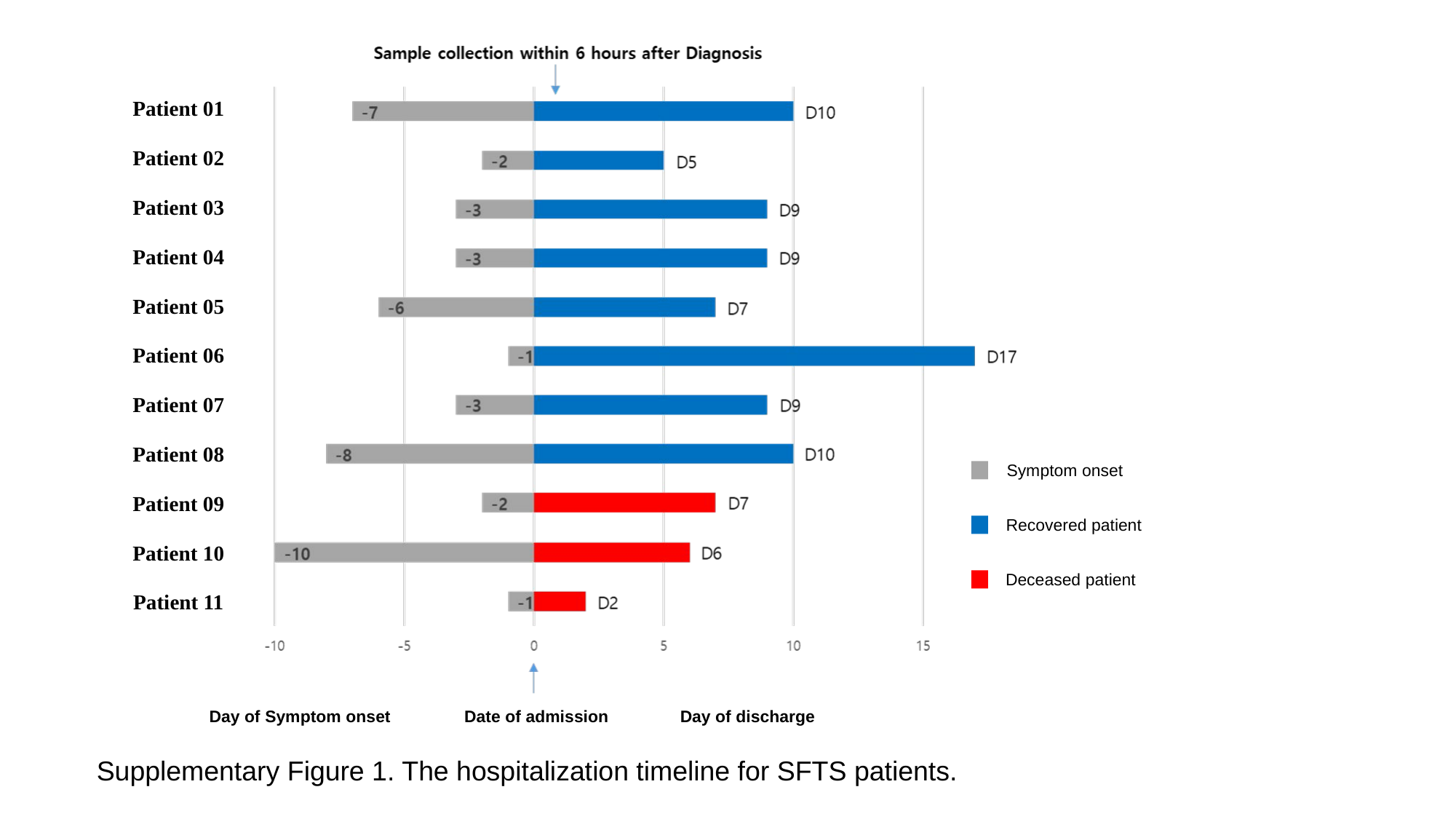

| Patient 01 |
| --- |
| Patient 02 |
| Patient 03 |
| Patient 04 |
| Patient 05 |
| Patient 06 |
| Patient 07 |
| Patient 08 |
| Patient 09 |
| Patient 10 |
| Patient 11 |
Symptom onset
Recovered patient
Deceased patient
Day of Symptom onset
Date of admission
Day of discharge
Supplementary Figure 1. The hospitalization timeline for SFTS patients.
